# Multiple Analytical Approaches Demonstrate a Complex Relationship of Genetic and Nongenetic Factors with Cisplatin- and Carboplatin-Induced Nephrotoxicity in Lung Cancer Patients

**DOI:** 10.1155/2014/937429

**Published:** 2014-08-28

**Authors:** H. Eugene Liu, Kuan-Jen Bai, Yu-Chen Hsieh, Ming-Chih Yu, Chun-Nin Lee, Jer-Hua Chang, Han-Lin Hsu, Pei-Chih Lu, Hsiang-Yin Chen

**Affiliations:** ^1^Department of Internal Medicine, Wan Fang Hospital, Taipei Medical University, 111 Hsing-Long Road, Section 3, Taipei 116, Taiwan; ^2^Graduate Institute of Clinical Medicine, College of Medicine, Taipei Medical University, 250 Wu-Hsing Street, Taipei 110, Taiwan; ^3^Department of Clinical Pharmacy, School of Pharmacy, Taipei Medical University, 250 Wu-Hsing Street, Taipei 110, Taiwan; ^4^Department of Internal Medicine, Shuang Ho Hospital, Taipei Medical University, 291 Jhongjheng Road, Jhonghe District, New Taipei City 235, Taiwan; ^5^Department of Pharmacy, Wan Fang Hospital, Taipei Medical University, 111 Hsing-Long Road, Section 3, Taipei 116, Taiwan

## Abstract

*Background*. Cisplatin and carboplatin cause nephrotoxicity by forming platinum-DNA adducts and lead to cell death. *Methods*. One-hundred and sixteen Taiwanese lung cancer patients who received cisplatin or carboplatin more than twice were recruited, and their genotypes were determined. The risk of renal dysfunction, injury to the kidney, failure of kidney function, loss of kidney function, and end-stage kidney disease (RIFLE) criteria were used to evaluate the occurrence of nephrotoxicity. A logistic regression, multiple regression with a classification and regression tree (CART), and the Framingham study risk score were used to analyze interactions between genetic and nongenetic factors in producing platinum-induced nephrotoxicity. *Results*. *ERCC1* 118C and *TP53* 72Arg polymorphisms were associated with increased risks of platinum-induced nephrotoxicity. Other risk factors found included the platinum type, baseline serum creatinine (Scr), coadministration of vinorelbine, and the number of chemotherapy cycles. The overall prediction rate of the CART was 82.7%, with a sensitivity of 0.630 and specificity of 0.896. The Framingham study risk prediction model contained 7 factors. Its prediction rate was 84.5%, with a sensitivity of 0.643 and specificity of 0.909. *Conclusions*. Genetic polymorphisms of *ERCC1* and *TP53* are risk factors for nephrotoxicity. The CART analysis may provide a clinically applicable model to predict the risk of cisplatin- and carboplatin-induced nephrotoxicity.

## 1. Introduction

Cisplatin and carboplatin are standard treatments for lung cancer. Unfortunately, these platinum-containing chemotherapies can induce severe nephrotoxicity, which limits their usage. Nephrotoxicity occurs in approximately one-third of cisplatin-treated patients, even after aggressive hydration [1]. Carboplatin is less nephrotoxic but still leads to elevated serum creatinine (Scr) in 10% of the patients [2]. Both cisplatin- and carboplatin-induced nephrotoxicity are dose-related. Patients who received more than 40 mg cisplatin/m^2^ per day or more than 1750 mg carboplatin are at higher risk of nephrotoxicity [3, 4]. Other identified risk factors include coadministration with nephrotoxic agents (e.g., ifosfamide), an older age, smoking, a female gender, and hypoalbuminemia [5–7].

Platinum binds to DNA to form platinum-DNA adducts, which induce cell death. Both tumor and renal cells are able to uptake large amounts of platinum. The concentration of cisplatin in proximal tubular cells was reported to be 5 times higher than that in serum [8]. The toxic effects of the adducts can be ameliorated by the nucleotide excision repair (NER) pathway [9]. Excision repair cross-complementing 1 (ERCC1) is a key enzyme which acts as a 5′ endonuclease to cleave platinum from DNA in the NER pathway. A polymorphism of* ERCC1* C118T was associated with a higher risk of developing cancer and negative clinical outcomes in lung cancer [10, 11]. However, the relationship between the* ERCC1* C118T polymorphism and platinum-induced nephrotoxicity is still inconclusive [2, 11–13].

As the gatekeeper of cell cycle or death, platinum-induced apoptosis is strongly regulated by tumor suppressor protein 53 (TP53) [9]. The importance of TP53 in platinum-induced nephrotoxicity was suggested by previous in vivo and in vitro studies. Cisplatin-induced cell apoptosis, the main cause of nephrotoxicity, was abrogated by pharmacological inhibitors, a dominant-negative mutation, and TP53 knockout [14–18]. The majority of cisplatin-activated TP53 accumulates in proximal tubules [14, 17]. A TP53 variant with a proline (Pro) at codon 72 had only one-fifth of the activity of inducing apoptosis compared to its wild-type (WT), which has arginine (Arg) at codon 72 [19]. However, the association between the TP53 Arg→Pro polymorphism and platinum-induced nephrotoxicity has not been studied.

The goals of this study were to examine the roles of ERCC1 and TP53 polymorphisms in platinum-induced nephrotoxicity and develop a prediction model which can identify patients susceptible to platinum-induced nephrotoxicity. The relationships of genetic and nongenetic factors with cisplatin- and carboplatin-induced nephrotoxicity were first assessed by a multivariable regression. Given that platinum-induced nephrotoxicity involves multiple factors and results from multivariable regressions are not easily explainable clinically, two other classification methods were also evaluated. The classification and regression tree (CART) analysis is a nonparametric statistical method, which manages multiple categorical and continuous variables at the same time to generate a tree-shaped classification model [20]. The CART analysis was applied in recent studies to predict cancer risk and explore interactions of multiple factors in carcinogenesis [21–25]. The Framingham study risk score is another classification method, which considers multiple factors in a point scheme [26]. The Framingham study risk score has frequently been used in cardiovascular research to predict the risk of cardiovascular events or death in patients [27–30]. In this study, the CART and Framingham study risk score methods were applied to identify subgroups of patients susceptible to cisplatin- or carboplatin-induced nephrotoxicity.

## 2. Patients and Methods

### 2.1. Patients and Clinical Specimens

This study recruited lung cancer patients who were admitted to Wan Fang Hospital, Taipei Medical University, between January 2005 and March 2011. Patients who had received more than two cycles of cisplatin- or carboplatin-containing chemotherapy for lung cancer and were aged 12~100 years at the time of diagnosis were eligible. Patients who were pregnant or infected by the human immunodeficiency virus (HIV) were excluded. Because of the known risk of nephrotoxicity, patients who were coadministrated ifosfamide with cisplatin or carboplatin were also excluded [6]. Peripheral blood and the clinical information of patients were collected after obtaining informed consent. The study protocol was approved by the Institutional Review Board (IRB) of Wan Fang Hospital.

### 2.2. Nephrotoxicity Assessment

Patients who met any level of risk of renal dysfunction, injury to the kidney, failure of kidney function, loss of kidney function, or end-stage kidney disease (RIFLE) criteria were defined as having cisplatin- or carboplatin-induced nephrotoxicity in this study [31]. An Scr increase to 1.5-fold of the baseline was defined as a risk of renal dysfunction; a 2.0-fold injury to the kidney, 3.0-fold failure of kidney function, and need for renal replacement therapy for more than 4 weeks as loss of kidney function; and need for dialysis for more than 3 months as end-stage kidney disease.

### 2.3. Determination of Genotypes

DNA from peripheral blood mononuclear cells was extracted by proteinase K digestion followed by the conventional phenol-chloroform method as previously described [32].

Genotypes of* TP53* codon 72 (rs1042522) were determined by polymerase chain reaction-restriction fragment-length polymorphism (PCR-RFLP). The PCR was performed in a total volume of 25 *μ*L, containing 0.2 mM dNTPs (Protech, Taiwan), 1 mM MgCl_2_ (Protech), 1x Taq buffer (Mg^+^-free) (Protech), 0.04 U Taq polymerase (Protech), 2 *μ*L primers (Protech), and 1 *μ*L DNA. Primers were 5′-GAAGACCCAGGTCCAGATGA-3′ (forward) and 5′-ACTGACCGTGCAAGTCACAG-3′ (reverse). Amplification was carried out under the following conditions: 1 cycle of 94°C for 3 min, followed by 35 cycles of 94°C for 30 s, 55°C for 30 s, and 72°C for 30 s, and 1 cycle of 72°C for 10 min, followed by cooling down to 4°C. The PCR product of* TP53* codon 72 was digested with BstUI (New England Biolabs), separated on a 2% agarose gel, and visualized by ethidium bromide staining.

Genotypes of* ERCC1 *codon 118 (rs11615) were determined by a 5′ nuclease assay (TaqMan). Each PCR mixture with a volume of 20 *μ*L contained 2x TaqMan Master Mix (Applied Biosystem), 40x TaqMan SNP Genotyping Assay Mix (Applied Biosystem), and 0.5 *μ*L DNA. Amplification conditions were 1 cycle of 95°C for 10 min, followed by 40 cycles of 92°C for 15 s and 60°C for 1 min. The fluorescence detection and PCR were carried out in an ABI Prism 7300 (Applied Biosystem).

For each SNP, 20 randomly selected samples (17%) were selected to be genotyped by direct sequencing in the ABI Prism 3100 (Applied Biosystem) again to validate the accuracies of the PCR-RFLP and 5′ nuclease assay. The accuracies of both methods were 100%.

### 2.4. Statistical Analysis

A two-tailed *P* < 0.05 was considered statistically significant in this study. The Hardy-Weinberg equilibrium of alleles at individual loci was evaluated. SPSS version 16.0 (SPSS, Chicago, IL, USA) for Windows was used to analyze data. Chi-squared and Fisher's exact tests were used to analyze ordinal and categorical data, and Student's* t*-test was used for continuous data. A multivariable logistic regression was applied to calculate the adjusted odd ratios (ORs) and 95% confidence intervals (CIs) of specific genotypes or variables of interest after adjusting for age, gender, body weight, alcohol consumption, smoking, baseline Scr, treatment regimen, number of chemotherapy cycles, and cancer histology.

The classification and regression tree (CART) and nephrotoxicity risk score system were performed to establish the most useful model to predict nephrotoxicity. Then the sensitivity, specificity, and total prediction rate of the CART and risk score models were compared. The Hosmer and Lemeshow method was also applied by using variables included in the CART and Framingham models to examine the goodness-of-fit with the Akaike information criterion (AIC) as an adjuvant parameter. Smaller values of the AIC indicate better models.

The CART based on binary recursive partitioning was used to explore gene-gene and gene-environment interactions [33]. Data were randomly divided into a training set (90% of the data) and a test set (10% of the data). The training set was used to build the tree, and the test set was used to estimate the accuracy of the built tree. The* Gini* index was used as a splitting criterion to stratify the data into various risk subgroups with maximum homogeneity to build the model. Tree-building continued until the terminal nodes had no statistically significant splits or reached a minimum size of 10 subjects for each terminal node. The ORs and 95% CIs of the terminal nodes were calculated using a logistic regression.

A nephrotoxicity risk score was established according to the statistical analysis method in the Framingham study, which used a point system to predict a patient's risk [26]. Variables included in the initial model were based on statistical considerations (*P* < 0.1) and biological relevance, such as smoking [7]. The initial score of each possible risk factor was estimated according to the regression coefficient in the logistic regression. The discriminatory power of the scale was determined by the area under the receiver operating characteristic curve (ROC), and Youden's index was used to identify the cut-off point to distinguish high or low risks of developing cisplatin-induced nephrotoxicity. Youden's index is defined as the maximum (sensitivity + specificity − 1) [34].

## 3. Results

### 3.1. Patients

Demographic characteristics of the 28 (24.1%) cases and 88 (75.9%) controls are listed in Table 1. There were no statistical differences in baseline patient characteristics, including gender, age, weight, alcohol consumption, and smoking, between cases and controls. The baseline Scr was also similar between cases and controls. The majority of patients (83, 71.6%) had received gemcitabine plus cisplatin/carboplatin. Patients with nephrotoxicity had received more cycles of chemotherapy than controls (4.96 ± 1.953 versus 4.16 ± 1.653, *P* = 0.034).

### 3.2. Genetic Variants

Alleles at individual loci fulfilled a Hardy-Weinberg distribution in both cases and controls (Table 1). In the multivariable regression, the variables with meaningful adjusted ORs (*P* > 0.1) were coadministration of vinorelbine, the number of chemotherapy cycles, and polymorphisms of ERCC1 C118T and TP53 Arg72Pro between cases and controls.

### 3.3. The CART

In an effort to transform gene-gene and gene-environment relationships to prediction systems that are convenient to use clinically, variables of characteristics and genetic polymorphisms were analyzed by two different statistical models.

**Figure 1:**
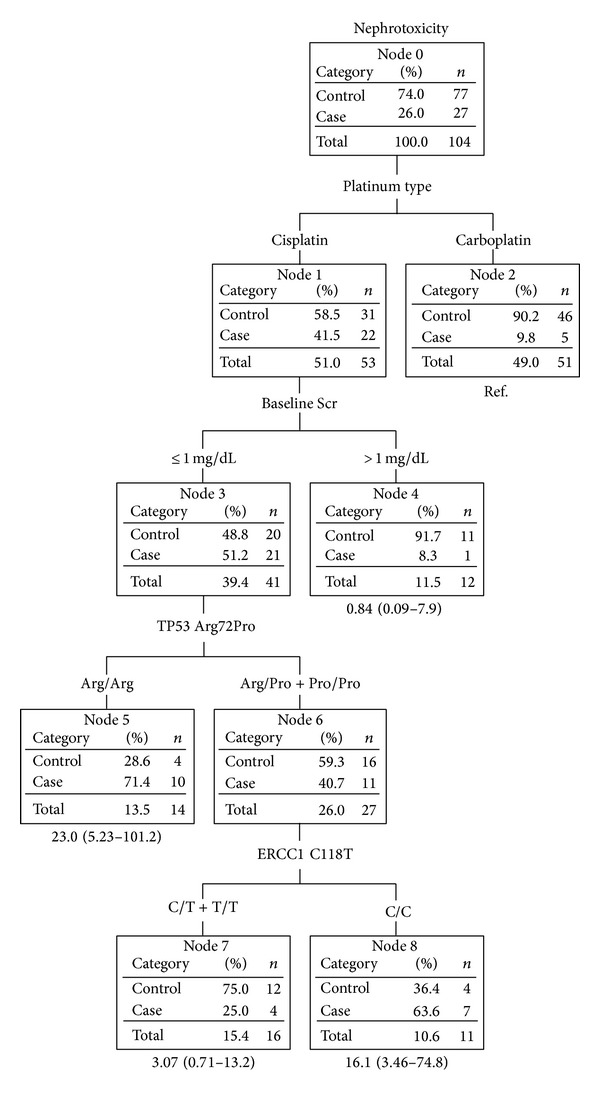
Classification and regression tree analysis of patients with and without platinum-induced nephrotoxicity.

The first evaluated model used a CART analysis. The decision tree of the CART is shown in Figure 1. The type of platinum therapy, baseline Scr, TP53 Arg72Pro genotype, and ERCC1 C118T were important factors for platinum-treated patients who experienced nephrotoxicity in the CART decision tree.

Subjects in node 8 who had received cisplatin with baseline Scr values of ≤1 mg/dL, the* TP53* Pro allele, and ERCC1 C/C and subjects in node 5 with the* TP53* Arg/Arg genotype were the subgroups most susceptible to nephrotoxicity. The case ratios were 71.4% and 63.6%, respectively. In contrast, subjects who received carboplatin (node 2) and those who received cisplatin with baseline Scr values of >1 mg/dL (node 4) had a lower nephrotoxicity risk, with respective case ratios of 9.8% and 8.3%. No interaction was found in patients treated with carboplatin due to a limited number of subjects with nephrotoxicity.

The overall prediction rate of the CART model was 82.7% (*n* = 86/104). Youden's index, the sensitivity, and specificity were 0.499, 0.630 (*n* = 17/27), and 0.869 (*n* = 69/77), respectively. Using the four variables identified by the CART, including the platinum type, baseline Scr,* TP53*, and* ERCC1, *the AIC value was 109.714.

### 3.4. Nephrotoxicity Risk Score

**Figure 2:**
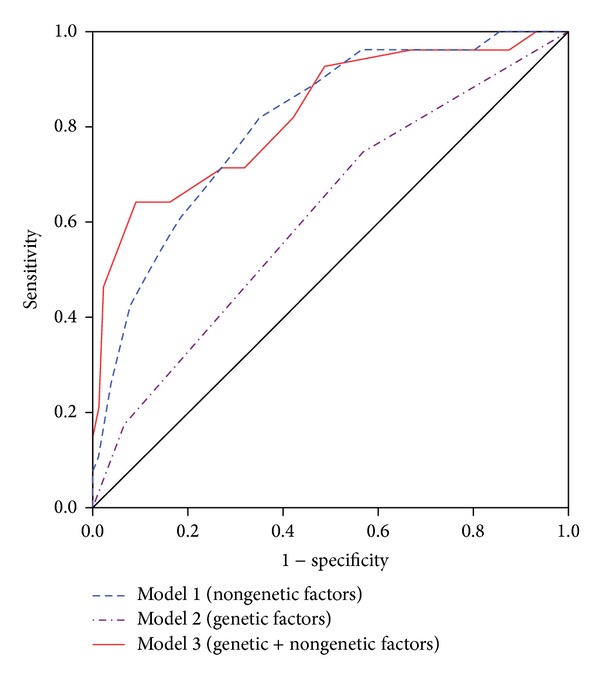
Receiver operating characteristic (ROC) curves from three models of a nephrotoxicity risk score.

**Figure 3:**
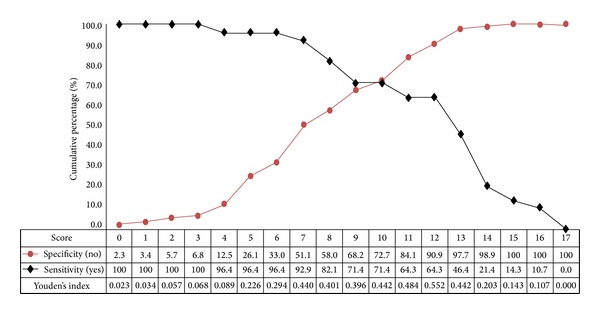
Sensitivity and specificity of the nephrotoxicity risk score of model 3 (genetic + nongenetic factors).

The second prediction model evaluated in this study was the risk score. The best model of the nephrotoxicity risk score is shown in Table 2. Three initial base models were compared: model 1 contained only environmental factors, including the type of platinum, baseline Scr, smoking status, coadministration of vinorelbine, and the number of chemotherapy cycles; model 2 contained only genetic factors, including TP53 Arg72Pro and ERCC1 C118T; and model 3 contained both genetic and environmental factors. The ROC curves from the 3 models are shown in Figure 2. The areas under the curves (AUCs) were 0.810, 0.616, and 0.829 for models 1, 2, and 3, respectively. Model 3 was the best model to predict cisplatin- or carboplatin-induced nephrotoxicity. Its overall prediction rate was 84.5% (*n* = 98/116). The sensitivity and specificity for each score are shown in Figure 3. The optimum cut-off point was 12 with the highest values for Youden's index (0.552), sensitivity (0.643), and specificity (0.909). The AIC for the multivariable regression was 108.869 using the 7 variables included in model 3.

## 4. Discussion

In the present study, we investigated the association of ERCC1 and TP53 gene polymorphisms with the risk of cisplatin- or carboplatin-induced nephrotoxicity by logistic regression and multifactor analytical approaches. No significant association of TP53 or ERCC1 with nephrotoxicity was detected by the multivariable regression. However, two multifactor analytical approaches generated different results.

Cisplatin, with 30% prevalence of nephrotoxicity, is known to be more nephrotoxic than carboplatin [35]. Therefore, it was not surprising that the type of platinum treatment was listed as one of the most important risk factors in our models. Interestingly, in the CART model, cisplatin-treated patients with a baseline Scr of ≤1 mg/dL were at higher risk of cisplatin-induced nephrotoxicity risk than those with a baseline Scr of >1 mg/dL, despite no difference in cumulative cisplatin doses between the groups (17.9 ± 6.39 versus 16.6 ± 5.49 mg/m^2^/wk). There are several possible explanations. It is possible that the renal function of patients with a baseline Scr of ≤1 mg/dL was usually overestimated. The cisplatin dose used in these patients may have been too high. Another possible reason may be inherent, such as TP53 and ERCC1, and can be explained by sequent nodes in the CART model. The relationship between baseline Scr and cisplatin-induced nephrotoxicity needs to be explored in future studies.

TP53 is an important protein that regulates cell cycle arrest and cell death, and variants with attenuated activity may therefore be less susceptible to drug-induced cell apoptosis [19]. Many in vivo and in vitro studies have explored the role of TP53 in cisplatin-induced nephrotoxicity [14–18]. The first study by Megyesi et al. showed an increased level of nuclear TP53 in rat kidneys after cisplatin treatment [36]. However, the role of TP53 in cisplatin-induced nephrotoxicity was not confirmed until a study by Cummings and Schnellmann [17]. Cummings and Schnellmann found that TP53 activation may be an early signal of cisplatin-induced renal apoptosis which was protected by pifithrin-α (a pharmacological inhibitor of TP53) [17]. Moreover, cisplatin-induced nephrotoxicity was also inhibited by a dominant-negative mutant of TP53 [18]. In mice, activation and accumulation of TP53 were determined by staining of both proximal and distal tubular cells which are recognized as primary sites of cisplatin-induced nephrotoxicity [14]. In addition, Wei et al. showed that TP53-WT mice demonstrated elevated Scr, which is a biomarker for kidney damage, compared to TP53-deficient mice after cisplatin treatment [14]. However, one clinical study of female patients indicated no association between TP53 Arg72Pro and cisplatin-induced nephrotoxicity [13].

Notably, an interesting finding of the TP53 polymorphism in our current CART model was that subjects with the TP53 mutation could be divided into two groups by the ERCC1 polymorphism. One group carrying the ERCC1 mutation had mildly increased Scr, but patients carrying the ERCC1 wild-type had obviously increased Scr. This phenomenon was also observed in an animal study reported by Wei et al. [14]. After cisplatin treatment, TP53-deficient mice could also roughly be divided into two groups. One group was resistant to cisplatin-induced renal injury and only showed mild to moderate increases in Scr. In contrast, the other group acted as TP53-WT mice with moderately to severely increased Scr which was seen in both TP53 and ERCC1 WT groups in our study [14]. Thus, ERCC1 may be an important split for cisplatin-treated patients who carry the TP53 mutation. ERCC1 is an important protein for repairing DNA damage. The present study is in agreement with previous reports on the role of the ERCC1 C118T polymorphism in cisplatin- and carboplatin-induced nephrotoxicity. The ERCC1 T allele has a protective effect against nephrotoxicity, with a significantly decreased risk of nephrotoxicity with the ERCC1 T allele [2, 13]. Developing different doses for patients with or without the ERCC1 T allele might prevent nephrotoxicity in lung cancer patients.

According to the nephrotoxicity risk score, simultaneously considering both genetic (TP53 and ERCC1) and nongenetic factors (baseline Scr ≤ 1 mg/dL, cisplatin, coadministration of vinorelbine, smoking, and treatment cycles > 4) can better predict the occurrence of platinum-induced nephrotoxicity than either type of factors alone. It was reported by de Jongh et al. that smoking may be a risk factor for cisplatin-induced nephrotoxicity [7]. However, no association was found between smoking and nephrotoxicity in the present study, which may have resulted from the small sample size of the present study that was about one-third of that of the study by de Jongh et al. Another nongenetic risk factor found in the present study was the number of chemotherapy cycles that patients received. Patients who received more chemotherapy cycles exhibited greater harm to the kidneys than those who received relatively fewer cycles. It was indicated that patients who had previously received cisplatin were at higher risk. However, we found that cisplatin-induced renal injury was shown with the same regimens rather than with different regimens. Other nongenetic risk factors, such as the coadministration of vinorelbine, need to be confirmed in future studies.

Both CART and risk score models demonstrated similar excellent predictive abilities (81.9% and 84.5%, resp.). Although the prediction rate of the risk score model was slightly higher than the CART model, more factors were used in the risk score than in the CART model (7 versus 4). Taking clinical usefulness into consideration, the CART model, containing fewer factors and a clinically similar prediction rate, might possess better clinical convenience than the risk score model.

Our CART model included only 4 factors (the type of platinum therapy, baseline Scr, TP53 Arg72Pro genotype, and ERCC1 C118T genotype) but still had a high overall prediction rate, sensitivity, and specificity (82.7%, 0.630, and 0.869, resp.). The CART analysis was applied to explore multifactor risks for various cancers, such as bladder cancer and oral premalignant lesion [21–23, 25]. The overall prediction rates of the CART models in those studies were medium (59.65%~66.13%), and the sensitivities and specificities of studies ranged 0.2~0.6. Numbers of nodes in these CART models of cancer studies were usually 5 or 6, with only 1 study including up to 13 factors.

Our study has several limitations. First of all, the sample size of our study was small. Some risk factors might not have been picked up by the statistical analysis due to the limited number of cases. For example, there were only 28 cases in 116 subjects and only 11 females with nephrotoxicity among the cases. Thus, sexual differences in the genetic effects might not have been indicated if they existed. Additionally, the range of the distribution of patient age was very wide, with 17 patients with more than 60 years. The small sample size also resulted in a large 95% CI in terminal nodes of the CART model that was split by 4 factors. In addition, no interaction was found in patients who received carboplatin because of a limited number of subjects who developed nephrotoxicity. Second, most of the patients (more than 70%) were subjected to combined treatment, such as cisplatin with gemcitabine, which can interfere in the nephrotoxicity. However, there was no statistically significant difference in the percentages of patients who were coadministered with gemcitabine in the case and control groups (Table 1). Third, patient data were retrospectively collected in charts of our hospital. Finally, we only investigated limited number of genes in our study. Other genes, such as OCT2, a channel protein that regulates platinum uptake into cells, may also play an important role in platinum-induced nephrotoxicity [37].

To sum up, the present study indicated that* TP53* Arg72Pro and* ERCC1* C118T play roles in cisplatin-induced nephrotoxicity. The study also demonstrated that classification statistical methods, such as the CART and risk score models, may be useful for evaluating risks of platinum-induced nephrotoxicity and develop dosing regimens for lung cancer patients. The results of this study need to be validated by future studies.

## Figures and Tables

**Table 1:** Comparisons between the cases and controls.

	Cases	Controls	OR^a^	95% CI	*P* value
	Number (%)	Number (%)			
Total	28	88			
Gender					0.96
Male	17 (60.7)	53 (60.2)			
Female	11 (39.3)	35 (39.8)			
Age (years)					
Mean ± SD	63.43 ± 9.47	66.66 ± 11.64			0.19
≥60	17 (60.7)	64 (72.7)			0.23
<60	11 (39.3)	24 (27.3)			
Weight (kg)					
Mean ± SD	59.6 ± 10.9	58.0 ± 9.78			0.48
Alcohol consumption	3 (10.7)	11 (12.5)			0.80
Smoking	13 (46.4)	36 (40.9)			0.61
Baseline Scr (mg/dL)					
Mean ± SD	0.83 ± 0.36	0.91 ± 0.32			0.22
Treatment regimen					
G + C/Cb	20 (71.4)	63 (71.6)			0.99
P/D + C/Cb	8 (28.6)	20 (22.7)			0.52
E + C/Cb	3 (10.7)	8 (9.1)			0.73
V + C/Cb	10 (35.7)	16 (18.2)			0.05
CCRT	2 (7.1)	5 (5.7)			0.68
Other	3 (10.7)	8 (9.1)			0.73
Number of cycles					
Mean ± SD	4.96 ± 1.95	4.16 ± 1.65			0.034∗
*ERCC1 C118T *					
CC	14 (27.5)	37 (72.5)	Ref		
CT	10 (18.2)	45 (81.8)	0.29	0.09~1.00	0.05
TT	4 (40.0)	6 (60.0)	0.77	0.15~3.93	0.76
CT + TT	14 (21.5)	51 (78.5)	0.37	0.12~1.14	0.08
*TP53 Arg72Pro *					
Arg/Arg	12 (38.7)	19 (61.3)	Ref		
Arg/Pro	12 (19.4)	50 (80.6)	0.38	0.12~1.26	0.11
Pro/Pro	4 (17.4)	19 (82.6)	0.34	0.07~7.44	0.18
Arg/Pro + Pro/Pro	16 (18.8)	69 (81.2)	0.37	0.12~1.17	0.09

SD: standard deviation; Scr: serum creatinine; C: cisplatin; Cb: carboplatin; G: gemcitabine; P: paclitaxel; D: docetaxel; E: etoposide; V: vinorelbine; CCRT: combined chemoradiotherapy.

^a^The odds ratio (OR) and 95% confidence interval (CI) were adjusted for age, gender, body weight, alcohol consumption, smoking, baseline Scr, treatment regimen, number of chemotherapy cycles, histology, and cancer type.

∗ *P* < 0.05.

**Table 2:** Nephrotoxicity risk score.

Parameter	*β* coefficient	OR	95% CI	Score
Nongenetic factors				
Baseline Scr ≤1 mg/dL	2.303	10.0	1.57~62.9	5
Cisplatin	1.790	5.99	1.53~23.5	4
Coadministration of vinorelbine	1.369	3.93	0.86~18.1	3
Smoking	0.528	1.69	0.41~7.05	2
Number of cycles >4	0.098	1.10	0.47~4.93	1
Genetic factors				
*TP53* wild-type	0.989	2.70	0.91~9.14	2
*ERCC1* C118T wild-type	0.990	2.70	0.90~8.35	2

OR: odds ratio; CI: confidence interval; Src: serum creatine.
